# Prediction of COVID-19 in-hospital mortality in older patients using artificial intelligence: a multicenter study

**DOI:** 10.3389/fragi.2024.1473632

**Published:** 2024-10-17

**Authors:** Massimiliano Fedecostante, Jacopo Sabbatinelli, Giuseppina Dell’Aquila, Fabio Salvi, Anna Rita Bonfigli, Stefano Volpato, Caterina Trevisan, Stefano Fumagalli, Fabio Monzani, Raffaele Antonelli Incalzi, Fabiola Olivieri, Antonio Cherubini

**Affiliations:** ^1^ Geriatria, Accettazione Geriatrica e Centro di ricerca per l’invecchiamento, IRCCS INRCA, Ancona, Italy; ^2^ Department of Clinical and Molecular Sciences, Università Politecnica Delle Marche, Ancona, Italy; ^3^ Clinic of Laboratory and Precision Medicine, IRCCS INRCA, Ancona, Italy; ^4^ Scientific Direction, IRCCS INRCA, Ancona, Italy; ^5^ Department of Medical Sciences, University of Ferrara, Ferrara, Italy; ^6^ Department of Experimental and Clinical Medicine, Geriatric Intensive Care Unit, University of Florence, Florence, Italy; ^7^ Intermediate Care Unit, Nursing Home Misericordia, Pisa, Italy; ^8^ Unit of Geriatrics, Department of Medicine, Campus Bio-Medico University and Teaching Hospital, Rome, Italy

**Keywords:** COVID-19, mobility, neutrophil-to-limphocyte ratio, in-hospital mortality, artificial intelligence

## Abstract

**Background:**

Once the pandemic ended, SARS-CoV-2 became endemic, with flare-up phases. COVID-19 disease can still have a significant clinical impact, especially in older patients with multimorbidity and frailty.

**Objective:**

This study aims at evaluating the main characteristics associated to in-hospital mortality among data routinely collected upon admission to identify older patients at higher risk of death.

**Methods:**

The present study used data from Gerocovid-acute wards, an observational multicenter retrospective-prospective study conducted in geriatric and internal medicine wards in subjects ≥60 years old during the COVID-19 pandemic. Seventy-one routinely collected variables, including demographic data, living arrangements, smoking habits, pre-COVID-19 mobility, chronic diseases, and clinical and laboratory parameters were integrated into a web-based machine learning platform (Just Add Data Bio) to identify factors with the highest prognostic relevance. The use of artificial intelligence allowed us to avoid variable selection bias, to test a large number of models and to perform an internal validation.

**Results:**

The dataset was split into training and test sets, based on a 70:30 ratio and matching on age, sex, and proportion of events; 3,520 models were set out to train. The three predictive algorithms (optimized for performance, interpretability, or aggressive feature selection) converged on the same model, including 12 variables: pre-COVID-19 mobility, World Health Organization disease severity, age, heart rate, arterial blood gases bicarbonate and oxygen saturation, serum potassium, systolic blood pressure, blood glucose, aspartate aminotransferase, PaO2/FiO2 ratio and derived neutrophil-to-lymphocyte ratio.

**Conclusion:**

Beyond variables reflecting the severity of COVID-19 disease failure, pre-morbid mobility level was the strongest factor associated with in-hospital mortality reflecting the importance of functional status as a synthetic measure of health in older adults, while the association between derived neutrophil-to-lymphocyte ratio and mortality, confirms the fundamental role played by neutrophils in SARS-CoV-2 disease.

## Introduction

On 5 May 2023, the World Health Organization officially declared the end of the SARS-CoV-2 health emergency that started 3 years earlier, on 11 March 2020. In these 3 years, according to World Health Organization (WHO) estimates, the SARS-CoV-2 virus caused about 20 million deaths and numerous other health and social problems. However, although the pandemic emergency is over, COVID-19 has not disappeared. Indeed, during the 2023-2024 winter, COVID-19 cases and hospitalization rates increased across the European Union (EU) (European Centre for Disease Prevention). Although many cases are asymptomatic, COVID-19 can still substantially increase in-hospital mortality, especially in older adults (Rizza et al., 2024). It is therefore crucial for clinicians to identify as early as possible those subjects who are at the highest risk of developing severe COVID-19 during hospitalization to optimize the use of health resources and to decrease the chance of fatal outcomes. Several studies on hospitalized patients affected by COVID-19 have been conducted with the aim of investigating demographics, clinical conditions and laboratory markers associated with severe disease outcomes (Wynants et al., 2020; Maestre-Muniz et al., 2022).

In 2020 Mendes et al. (2020) conducted a retrospective cohort study on 235 older Caucasian patients of mean age 86 ± 6.5 years, considering demographics, clinical, imaging and few routine laboratory parameters to determine predictors of in-hospital mortality related to COVID-19 in older patients. Using logistic regression and Cox proportional hazard models to predict mortality they found that male sex, crackles, a higher fraction of inspired oxygen, and functionality were independent risk factors for in-hospital mortality. In a post-hoc analysis on 1,520 patients aged ≥65 years from the HOPE COVID-19 registry, the authors found that age ≥75 years, dementia, low peripheral oxygen saturation, severe lymphopenia and qSOFA scale >1 were independent predictors of in-hospital mortality (Becerra-Muñoz et al., 2021). In a single-centre prospective study on 239 older patients (median age 85 years), Covino et al. (2021) found that regardless of disease severity, increasing age, dementia, and impairment in activities of daily living (ADL) were strong risk factors for in-hospital mortality. Finally, Ramos-Rincon et al. in a multicenter, retrospective, observational study on hospitalized COVID-19 older adults confirmed the possible relevance of preadmission clinical status on in-hospital mortality beyond parameters related to disease severity (Ramos-Rincon et al., 2021). Since the beginning of the pandemic, numerous evidence has also accumulated on the possible role of neutrophils in the severity of COVID-19 (Wu et al., 2020).

More specifically, a higher neutrophil-to-lymphocyte ratio (NLR) has been shown to predict mortality in hospitalized older adults (Di Rosa et al., 2023) and in COVID-19 patients (Alkhatip et al., 2021). Neutrophil-to-lymphocyte ratio was related to in-hospital mortality in a Spanish cohort of 177 hospitalized COVID-19 older patients with a World Health Organization ordinal scale 4 (oxygen by masque or nasal prongs) or 5 (non-invasive ventilation or high-flow oxygen) (Lozano et al., 2022). In a previous work we also found that, in geriatric patients admitted to hospital for COVID-19, beyond age, laboratory markers at admission, such as high neutrophil percentage and NLR, were among the best and independent predictors of in-hospital mortality (Olivieri et al., 2022). The predictive models vary in their results on the basis of different variables considered and could be biased by a pre-selection of variable to be included in multivariate models. Machine learning and artificial intelligence algorithms can overcome these issues (Wendland et al., 2023) and potentially enhance the predictive capabilities of models developed with traditional statistics (Riela, 2023). Casas-Rojo et al. (2023), using machine learning techniques to develop predictive models of mortality in patients with COVID-19 from the SEMI-COVID-19 registry at hospital admission, found that the model developed with machine learning technique has a better predictive capacity than a previous model developed on the same population using traditional statistical methods. In this study, using a web-based auto-machine-learning platform and an artificial intelligence decision support system, we aim to identify the main factors associated with in-hospital mortality among several clinical, anamnestic, and laboratory data routinely collected at admission in a cohort of hospitalized older adults in different Italian hospitals.

## Materials and methods

### Study design and participants

The present study used data of 819 patients from the Gerocovid-acute wards substudy, an observational multicenter retrospective-prospective initiative, enrolling individuals aged ≥60 years either retrospectively or prospectively, conducted in geriatric and internal medicine wards in older subjects who had been confirmed to be infected with SARS-CoV-2 by real-time reverse transcriptase-polymerase chain reaction assay regardless of the clinical symptoms. The enrollment started on 1 March 2020 and ended on 31 December 2020, with a follow-up until 30 June 2021. A complete description of the study methodology has been previously published (Trevisan et al., 2021; Okoye et al., 2022; Coin et al., 2023). The study was conducted following the STROBE guidelines. Data registration was performed using a dedicated electronic register designed by Bluecompanion (UK, France) to collect all clinical data from every investigational site across Italy. The primary outcome was in-hospital mortality, defined as death during the hospitalization.

### Ethics statement

The study protocol has been approved by the Ethics Committee of the Campus Bio-Medico University (reference number 22/20 OSS) and registered under the ClinicalTrials.gov database (reference number NCT04379440). Each study site’s ethics committee approved the protocol. All statistical analyses were performed on anonymized data. All research was performed in accordance with relevant guidelines and regulations.

### Data collection and preprocessing

At hospital admission, GeroCovid researchers collected data concerning the demographic data (sex, age, ethnicity), living arrangements, smoking habits, and pre-COVID-19 mobility (categorized as moving independently, using walking aid/moving with a wheelchair, moving with assistance in a wheelchair/bedridden). The presence of chronic diseases was retrieved from medical charts, in particular arterial hypertension, cardiovascular diseases (including cardiomyopathies, ischemic heart disease, heart failure, atrial fibrillation), chronic obstructive pulmonary disease (COPD), diabetes mellitus, obesity, chronic renal failure, depression, and cognitive impairment.

All laboratory biomarkers, including arterial blood gas analysis, complete blood count, albumin, glucose, potassium, sodium, chloride, blood urea nitrogen (BUN), creatinine, aspartate aminotransferase (AST), alanine aminotransferase (ALT), gamma-GT, total bilirubin, lactate dehydrogenase (LDH), high-sensitivity CRP (hs-CRP), d-dimer, procalcitonin, INR, aPTT, and fibrinogen, were measured by standard procedures.

Variables with <40% of missing values were included in the dataset as predictors. Multiple imputation was performed on missing values using the package *mice* (van Buuren and Groothuis-Oudshoorn, 2011), assuming they were missing at random. No missing data on the primary outcome of in-hospital mortality were present. Data balancing was not performed in this study to preserve the real-world distribution of outcomes, which is inherently imbalanced in COVID-19 mortality datasets.

### Statistical analysis

Variables were summarized using descriptive statistics. Median and interquartile ranges were used for continuous variables, and frequencies and proportions for categorical measures. Mann–Whitney U and Chi-squared tests were used to evaluate differences between groups.

Just Add Data Bio (JADBIO), a web-based auto-machine-learning platform for analyzing potential biomarkers (Tsamardinos et al., 2022), was used. The platform employs a multivariate analysis approach to (i) identify the minimal set of features necessary for predicting a specific outcome, (ii) develop the optimal predictive model using those selected features, and (iii) evaluate the model’s performance. To ensure unbiased performance estimation, it uses Bootstrap Bias Corrected Cross-Validation (BBC-CV), which accounts for the testing of multiple machine learning pipelines. The classification methods include linear, ridge, and Lasso regression, decision trees, random forests (RF), and support vector machines (SVMs) with both Gaussian and polynomial kernels. To create a simple and interpretable model, the platform uses Statistically Equivalent Signatures (SES) for feature selection (Tsagris and Tsamardinos, 2018). Machine learning techniques such as penalized Cox regression, survival decision trees, and survival random forests are employed to build the predictive models. Each stage of the analysis is cross-validated to ensure fair performance evaluation of the models, with bootstrapping added to eliminate any optimism bias from overfitting.

Here, we tested configurations optimized for different criteria, namely, performance, interpretability, and aggressive feature selection. The Performance-optimized model reports the configuration with the highest expected predictive performance. The interpretability-optimized model produces the best-performing configuration among those whose predictive algorithm generates models that are humanly interpretable. The Aggressive Feature Selection model enforces the identification of minimal size feature subsets at the expense of reduced performance, on average.

JADBIO 1.4.93 with extensive tuning effort and 6 CPU was used to model the dataset on the overall 82 variables by splitting data into a training set and a test set in a 70:30 ratio. The training set was used for model training and, the test set was used for model evaluation. The outcome was in-hospital mortality. Model performance was assessed through the Harrell’s concordance index (c-index). The c-index computes the percentage of patient-pairs correctly ordered by the predictive algorithm according to their time-to-event. Censored cases are dealt with by removing the corresponding pair whenever a meaningful comparison in terms of time-to-event is not possible. A c-index of 1 indicates perfect ranking of their patients according to their relative risk while 0.5 indicates random risk assessment, and a value <0.5 corresponds to a model performing worse than random guessing.

The resulting model can be obtained upon request to the Corresponding Author and run with Java executor for the classification of COVID-19 samples based on the variables presented in the results for further explorations.

Statistical analysis was performed using R version 4.1, and Jamovi version 2.3. A two-sided *p* < 0.05 was regarded as statistically significant for all tests.

## Results

### Baseline subject characteristics

A total of 819 geriatric patients (mean age 78.5 ± 9.5) who were hospitalized at 19 investigational sites due to COVID-19 were included in the analysis. The 26% (n = 213) of the enrolled patients died during the in-hospital stay. The mean number of days from hospital admission to discharge for the recovered patients was 22.1 ± 17.8, and that for the deceased patients was 14.2 ± 13.1. The minimum number of days for which patients in the recovered group remained hospitalized was 1 day, while the maximum number was 97 days for survived patients and 76 days for deceased patients.

The clinical and laboratory characteristics of the study cohort at admission are reported in Table 1. Deceased patients were significantly older than survivors, whereas no sex-related difference was highlighted in terms of mortality. The prevalence of impaired mobility, including individuals capable of walking with a device and those confined in bed, and poor nutritional status were significantly higher among deceased patients. Overall, deceased patients had worse general conditions at admission and were characterized by a higher prevalence of cardiac (cardiomyopathy, atrial fibrillation, heart failure), central nervous system (CNS), renal, and autoimmune comorbidities. Regarding the laboratory assessments, deceased patients had significantly lower levels of hemoglobin and platelets, higher neutrophil counts, and consistently lower counts of the other leukocyte populations.

**TABLE 1:** Clinical and laboratory characteristics of the study cohort at admission.

Variable	Deceased N = 213	Survived N = 606	Total N = 819	p
Age (years)	84 (79–88)	77 (70–84)	79 (71–86)	<0.001
Sex (males, %)	114 (53.5)	296 (48.8)	410 (50.1)	0.274
*Smoking status (n, %)*				
No	133 (62.4)	415 (68.5)	548 (66.9)	0.030
Yes	3 (1.4)	22 (3.6)	25 (3.1)	
Unknown	77 (36.2)	169 (27.9)	246 (30.0)	
*Drinking (n, %)*				
No	78 (36.6)	249 (41.1)	327 (39.9)	0.088
Yes	10 (4.7)	48 (7.9)	58 (7.1)	
Unknown	125 (58.7)	309 (51.0)	434 (53.0)	
*Mobility classification (n, %)*				
Can walk independently	61 (28.6%)	383 (63.2%)	444 (54.2%)	<0.001
Can walk with a cane	24 (11.3%)	52 (8.6%)	76 (9.3%)	
Can walk with a walker	37 (17.4%)	69 (11.4%)	106 (12.9%)	
Can move around with a wheelchair	10 (4.7%)	18 (3.0%)	28 (3.4%)	
Does not move around but is accompanied outside on the wheelchair	13 (6.1%)	17 (2.8%)	30 (3.7%)	
Is confined at home, mostly lying on the bed, sometimes sitting on the wheelchair	34 (16.0%)	38 (6.3%)	72 (8.8%)	
Lying on the bed, does not stand up or get in sitting position autonomously	34 (16.0%)	29 (4.8%)	63 (7.7%)	
*General condition (n, %)*				
Good	28 (13.1%)	307 (50.7%)	335 (40.9%)	<0.001
Bad	114 (53.5%)	260 (42.9%)	374 (45.7%)	
Very deteriorated	62 (29.1%)	37 (6.1%)	99 (12.1%)	
Terminal	9 (4.2%)	2 (0.3%)	11 (1.3%)	
*WHO disease severity (n, %)*				
No oxygen therapy (4)	31 (14.6%)	219 (36.1%)	250 (30.5%)	<0.001
Needs oxygen by mask or nasal prongs (5)	95 (44.6%)	269 (44.4%)	364 (44.4%)	
Severe disease, needs non-invasive and mechanical ventilation (6–9)	87 (40.9%)	118 (19.5%)	205 (25.0%)	
*Comorbidities (n, %)*				
Arterial hypertension	154 (72.3%)	415 (68.5%)	569 (69.5%)	0.298
Cardiomyopathy (ischemic, valvular, arrhythmias)	92 (42.3%)	187 (30.9%)	279 (34.1%)	0.001
Atrial fibrillation	56 (26.3%)	110 (18.2%)	166 (20.3%)	0.011
Peripheral artery disease	33 (15.5%)	67 (11.1%)	100 (12.2%)	0.089
Heart failure	46 (21.6%)	56 (9.2%)	102 (12.5%)	<0.001
History of stroke	26 (12.2%)	55 (9.1%)	81 (9.9%)	0.188
Diabetes	58 (27.2%)	159 (26.2%)	217 (26.5%)	0.778
Depression	22 (10.3%)	57 (9.4%)	79 (9.6%)	0.695
Osteoarthrosis	39 (18.3%)	129 (21.3%)	168 (20.5%)	0.355
COPD	42 (19.7%)	81 (13.4%)	123 (15.0%)	0.026
Chronic renal failure	48 (22.5%)	66 (10.9%)	114 (13.9%)	<0.001
Chronic liver disease	4 (1.9%)	13 (2.1%)	17 (2.1%)	0.814
Obesity	32 (15.0%)	81 (13.4%)	113 (13.8%)	0.546
Poor nutritional status	35 (16.4%)	45 (7.4%)	80 (9.8%)	<0.001
Dementia	105 (49.3%)	203 (33.5%)	308 (37.6%)	<0.001
Cancer	53 (24.9%)	118 (19.5%)	171 (20.9%)	0.095
Immune system disorders	9 (4.2%)	10 (1.7%)	19 (2.3%)	0.032
Number of comorbidities	4 (2–5)	3 (1–4)	3 (2–5)	<0.001
BMI (kg/m^2^)	25.0 (22.1–28.3)	25.4 (22.5–29.0)	25.4 (22.3–28.8)	0.302
Heart rate (bpm)	85.0 (74.0–100.0)	80.0 (70.0–90.0)	80.0 (70.0–90.0)	<0.001
Systolic BP (mmHg)	120.0 (110.0–135.0)	130.0 (120.0–140.0)	130.0 (115.0–140.0)	<0.001
Diastolic BP (mmHg)	70.0 (60.0–80.0)	70.0 (66.5–80.0)	70.0 (63.5–80.0)	<0.001
*Arterial blood gas*				
pH	7.5 (7.4–7.5)	7.5 (7.4–7.5)	7.5 (7.4–7.5)	0.034
PaO_2_ (mmHg)	64.0 (54.0–79.0)	70.0 (60.0–86.0)	69.0 (59.0–85.0)	<0.001
SpO_2_ (%)	94.0 (90.0–97.0)	95.0 (93.0–98.0)	95.0 (92.0–98.0)	<0.001
PaCO_2_ (mmHg)	34.0 (30.0–40.0)	35.0 (32.0–39.0)	35.0 (31.0–39.0)	0.115
HCO_3_- (mEq/L)	24.0 (22.0–27.0)	25.0 (24.0–28.0)	25.0 (23.0–28.0)	<0.001
P/F ratio	238.1 (147.6–304.8)	296.2 (231.1–360.7)	285.7 (204.8–347.6)	<0.001
*Laboratory*				
RBC (n x mm3)	4.2 (3.8–4.7)	4.3 (3.9–4.7)	4.3 (3.9–4.7)	0.075
Hemoglobin (g/dL)	12.4 (10.6–13.7)	12.6 (11.4–13.9)	12.6 (11.2–13.9)	0.021
Hematocrit (%)	37.4 (33.0–41.9)	38.1 (35.0–41.8)	38.0 (34.5–41.8)	0.129
MCV (fl)	89.7 (86.0–94.3)	89.7 (86.2–93.2)	89.7 (86.1–93.6)	0.592
MCH (pg)	29.6 (27.8–31.1)	29.7 (28.1–31.0)	29.6 (28.0–31.0)	0.714
MCHC (g/dL)	33.0 (32.0–34.1)	33.4 (32.4–34.3)	33.3 (32.3–34.3)	0.002
PLT (x10^3/mm3)	196.0 (143.0–257.0)	211.5 (159.2–271.8)	207.0 (155.0–269.5)	0.020
WBC (x10^3/mm3)	7.3 (5.8–10.2)	6.4 (4.7–8.7)	6.6 (4.9–9.0)	<0.001
Neutrophil %	83.4 (74.2–89.1)	74.0 (65.0–82.9)	77.0 (67.0–85.1)	<0.001
Lymphocyte %	9.6 (6.0–16.2)	16.0 (10.0–23.2)	14.1 (8.2–21.0)	<0.001
Monocyte %	5.6 (3.3–7.4)	7.5 (5.0–10.0)	6.8 (4.6–9.5)	<0.001
Eosinophil %	0.1 (0.0–0.4)	0.2 (0.0–0.8)	0.1 (0.0–0.7)	<0.001
Basophil %	0.2 (0.1–0.4)	0.2 (0.1–0.4)	0.2 (0.1–0.4)	0.004
Neutrophil #	6.0 (4.4–8.6)	4.6 (3.1–6.8)	5.0 (3.3–7.2)	<0.001
Lymphocyte #	0.7 (0.5–1.0)	1.0 (0.7–1.3)	0.9 (0.6–1.3)	<0.001
Monocyte #	0.2 (0.0–0.4)	0.3 (0.1–0.5)	0.2 (0.1–0.5)	<0.001
Eosinophil #	0.0 (0.0–0.0)	0.0 (0.0–0.1)	0.0 (0.0–0.1)	0.012
Basophil #	0.0 (0.0–0.0)	0.0 (0.0–0.0)	0.0 (0.0–0.0)	0.178
NLR	8.7 (4.6–14.7)	4.6 (2.7–7.7)	5.2 (3.1–10.1)	<0.001
dNLR	4.9 (2.8–8.2)	2.9 (1.8–4.7)	3.3 (2.0–5.7)	<0.001
PLR	253.1 (165.3–398.0)	215.4 (148.9–329.7)	225.0 (152.6–352.5)	0.002
LMR	4.1 (1.8–14.5)	3.8 (1.8–11.8)	3.9 (1.8–12.3)	0.245
Albumin (g/dL)	3.1 (2.7–3.4)	3.2 (2.8–3.5)	3.1 (2.8–3.5)	0.014
Glucose (mg/dL)	126.0 (102.0–158.2)	110.0 (95.0–132.1)	113.0 (96.0–139.0)	<0.001
Potassium (mEq/L)	4.0 (3.7–4.4)	4.0 (3.6–4.3)	4.0 (3.6–4.3)	0.016
Sodium (mEq/L)	138.0 (135.0–142.0)	138.0 (135.0–140.0)	138.0 (135.0–141.0)	0.102
Chloride (mEq/L)	101.0 (97.0–107.0)	100.0 (97.0–104.0)	100.0 (97.0–104.0)	0.017
BUN (mg/dL)	37.5 (17.5–73.0)	30.0 (15.0–50.9)	32.0 (15.0–54.0)	0.001
Creatinine (mg/dL)	0.8 (0.0–1.3)	0.8 (0.5–1.1)	0.8 (0.0–1.1)	0.814
AST (IU/L)	41.0 (30.0–58.0)	32.0 (21.0–46.0)	34.0 (23.0–50.0)	<0.001
ALT (IU/L)	24.0 (16.0–43.0)	23.0 (16.0–39.8)	23.0 (16.0–40.0)	0.156
Gamma-GT (IU/L)	36.0 (22.0–74.0)	32.0 (19.0–59.8)	33.0 (20.0–61.0)	0.024
Total bilirubin (mg/dL)	6.8 (0.5–12.0)	6.8 (0.6–12.0)	6.8 (0.6–12.0)	0.440
LDH (IU/L)	343.0 (240.0–504.0)	287.0 (218.2–410.5)	300.0 (223.0–438.0)	<0.001
hs-CRP (mg/L)	87.5 (19.3–162.1)	35.3 (7.0–104.0)	43.0 (8.6–119.5)	<0.001
D-dimer (mg/L)	1,006.0 (549.0–2,920.0)	830.0 (463.5–1,527.0)	896.0 (467.5–1782.0)	<0.001
Procalcitonin (ng/mL)	0.3 (0.1–0.7)	0.1 (0.1–0.2)	0.1 (0.1–0.3)	<0.001
aPTT ratio	1.1 (0.9–1.2)	1.0 (0.9–1.1)	1.0 (0.9–1.2)	0.593
INR	1.2 (1.1–1.3)	1.2 (1.1–1.3)	1.2 (1.1–1.3)	0.023
Fibrinogen (mg/L)	5,564.8 (3,401.4–7,242.4)	5,599.5 (3,609.6–7,172.9)	5,587.9 (3,551.8–7,172.9)	0.707

BP, blood pressure; COPD, chronic obstructive pulmonary disease; INR, international normalized ratio; LMR, lymphocyte-to-monocyte ratio; NLR, neutrophil-to-lymphocyte ratio; dNLR, derived NLR; PLR, platelet-to-lymphocyte ratio; RBC, red blood cells; WBC, white blood cells.

### Artificial intelligence-based in-hospital mortality prediction

**FIGURE 1:**
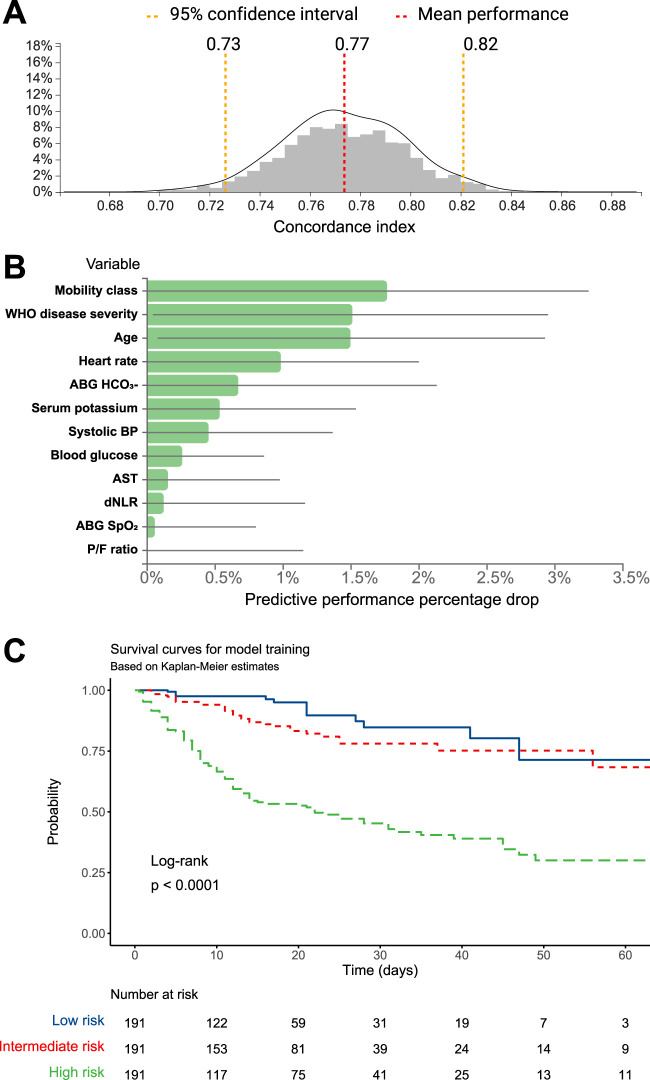
**(A)** C-statistic distributions and **(B)** predictive performance drop for the mortality prediction model computed on the training dataset. **(C)** Kaplan-Meier survival estimates for the training dataset, stratified according to categories of mortality risk.

The dataset was split into training and test sets based on a 70:30 ratio and matching on age, sex, and proportion of events. The JADBIO’s AI system estimated the out-of-sample performance of the models produced by each configuration using Repeated 10-fold CV without dropping (max. repeats = 20). Overall, 176 configurations × 20 repeats × 10 folds = 3,520 models were set out to train. Three predictive algorithms optimized for performance, interpretability, or aggressive feature selection, were computed (details in Methods). Of notice, the three algorithms converged on the same model, computed using Ridge Cox Regression with penalty hyper-parameter lambda = 1.0. The model achieved a good predictive ability, with a concordance index of 0.774 (95% CI 0.726–0.821) (Figure 1A). The complete analysis report is available at JADBio website (JADBio, 2024).

The prediction algorithm selected 12 variables out of the 71 available variables. Variables included in the model, along with the model coefficients and derived odds ratios (OR) are reported, in order of decreasing importance, in Table 2.

**TABLE 2:** Model coefficient for each predictor. A positive coefficient indicates that as the value of the independent variable increases, the mortality risk also increases.

Order of importance	Feature	Coefficient	Exp(Coefficient)
1	Pre-COVID-19 mobility		
	Walks independently	Ref.	Ref.
	Walks with the aid of ambulatory devices (cane, walker)	0.724	2.063
	Reduced mobility (on wheelchair, mostly or totally on bed)	0.969	2.635
2	WHO disease severity classification		
	Hospitalised; no oxygen therapy (4)	Ref.	Ref.
	Hospitalised; oxygen by mask or nasal prongs (5)	0.327	1.386
	Hospitalised: severe disease (6–9)	0.992	2.698
3	Age	0.339	1.403
4	Heart rate	0.222	1.248
5	ABG HCO_3_^−^	−0.300	0.741
6	Serum potassium	0.164	1.178
7	Systolic blood pressure	−0.151	0.860
8	Blood glucose	0.064	1.067
9	AST	0.135	1.145
10	dNLR	0.190	1.209
11	ABG SpO_2_	−0.128	0.879
12	P/F ratio	−0.264	0.768

ABG, arterial blood gas bicarbonate; AST, aspartate aminotransferase; dNLR, derived neutrophil-to-lymphocyte ratio.

The training model included age, pre-COVID-19 mobility, WHO disease severity, and variables that are routinely assessed upon hospital admission in COVID-19 patients, such as the ABG-derived parameters SpO_2_, HCO_3_^−^, and the P/F ratio, the vital signs systolic BP and heart rate, levels of blood glucose, aspartate aminotransferase (AST), and the derived neutrophil-to-lymphocyte ratio (dNLR, calculated as neutrophil count divided by the result of white blood cells (WBC) count minus neutrophil count).

The plots in Figure 1B show the predictive performance percentage drop that will result from removing specific variables from the model.

The model was used by the algorithm to estimate the mortality probability for each patient in the training group. Patients were then grouped based on tertiles of predicted mortality into high-, intermediate-, and low-risk. Kaplan-Meier survival functions, performed to illustrate differences in mortality according to the models’ predictions, showed that the training model achieved a significant stratification of patients according to the mortality risk (log-rank *p* < 0.001, Figure 1C). The Cox regression model, computed using the probability tertile as a predictor, confirmed the increasing trend of hazard ratios among groups, which were significantly different from each other (*p* < 0.001, Table 3).

**TABLE 3:** Survival statistics and Cox regression for in-hospital mortality prediction in the training dataset.

Risk category	n	Events	Mean survival (days)	SE	Median survival (days)	HR (95% CI)	HR (95% CI)
Low	191	13	78.3	5.7	n.a	Ref.	-
Intermediate	191	33	67.0	6.2	76.0	2.08 (1.10–3.96)	Ref.
High	191	103	40.7	3.6	22.0	7.48 (4.20–13.32)	3.61 (2.44–5.35)

n.a. not applicable.

**FIGURE 2:**
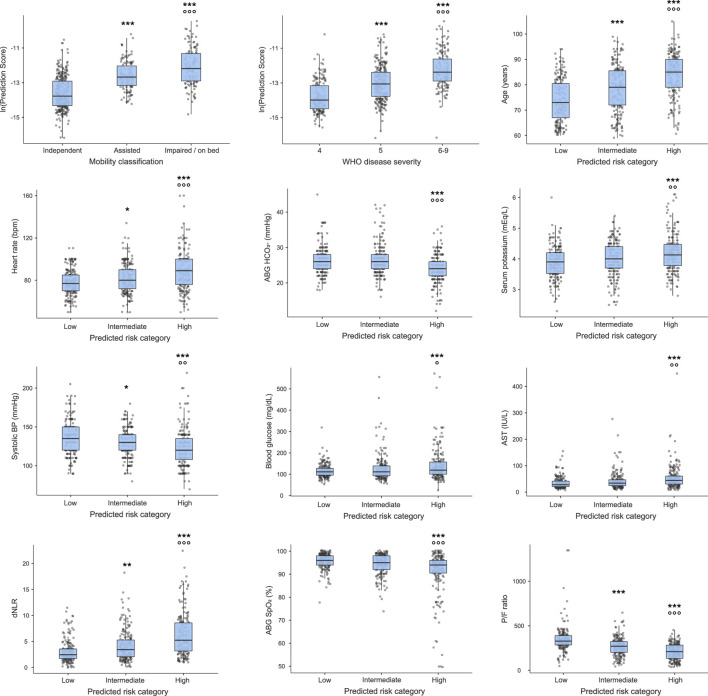
Risk prediction scores for the 12 predictors included in the training model. Figure legend: *p < 0.05, **p < 0.01, ***p < 0.001 vs. low-risk, walks independently, or WHO disease severity class 4; *p < 0.05, **p < 0.01, ***p < 0.001 vs. intermediate-risk, assisted mobility or WHO disease severity class 5 for Dunn’s post-hoc analysis.

Significant differences among groups were highlighted in the distribution of all the variables included in the model (Figure 2). In particular, WHO disease severity, age, heart rate, and dNLR significantly increased from low-to intermediate-to high-risk patients, whereas systolic BP and the P/F ratio followed an opposite trend. Serum potassium, blood glucose, and AST were higher, whereas HCO3- and SpO2 were lower in high-risk compared to intermediate- and low-risk patients. Moreover, a progressive increase in the predicted mortality risk was observed with deteriorating pre-COVID-19 mobility.

**FIGURE 3:**
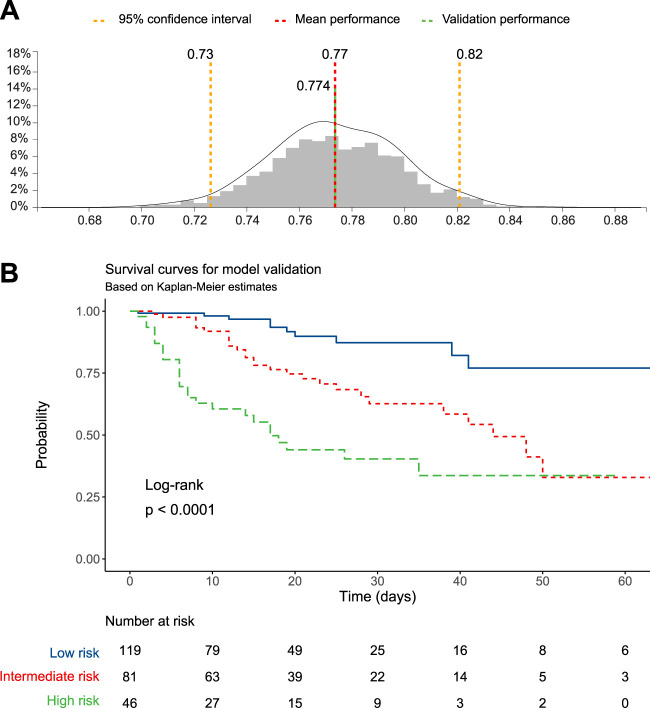
Model validation: **(A)** C-statistic (in green), **(B)** Kaplan-Meier survival function.

Finally, the training model was validated against the test set, composed of 30% cases of the original dataset. The model maintained the performance achieved during training, with a validation concordance index of 0.774 (Figure 3A). Therefore, the survival function was computed for patients grouped according to tertiles of predicted mortality. The Kaplan-Meier survival function was statistically significant (log-rank *p* < 0.0001, Figure 3B), and the Cox regression computed using tertiles of predicted probability as predictors confirmed that the high-risk group had a higher mortality risk compared to the low- and intermediate-risk groups (Table 4).

**TABLE 4:** Survival statistics and Cox regression for in-hospital mortality prediction in the test set.

Risk category	n	Events	Mean survival (days)	SE	Median survival (days)	HR (95% CI)	HR (95% CI)
Low	119	10	77.1	4.6	n.a	Ref.	-
Intermediate	81	28	43.3	3.9	44.0	3.80 (1.84–7.86)	Ref.
High	46	26	39.0	6.7	17.0	8.54 (4.08–17.88)	2.26 (1.32–3.88)

n.a. not applicable.

## Discussion

In this study, from 71 available variables, including sociodemographic data, smoking habits, mobility, chronic diseases, and clinical and laboratory parameters, we identified 12 factors associated with in-hospital mortality in older inpatients with SARS-CoV-2 infection. These selected were able to detect with moderate accuracy patients at increased risk of in-hospital mortality.

Using a statistical analysis method based on machine learning and artificial intelligence allowed us to test all the variables collected during the study, avoiding variable selection bias. AI models have the advantage of capturing more complex, and not always linear, relationships between variables, offering the possibility to test several models (Ngiam and Khor, 2019). Here, routinely collected laboratory and clinical hospital admission data were integrated into a web-based machine learning platform to identify, among factors with established or putative association with unfavorable COVID-19 outcomes, those with the highest prognostic relevance in older patients.

In the final model, factors associated with a high risk of in-hospital mortality were pre-COVID-19 mobility, WHO disease severity, age, heart rate, ABG HCO3-, serum potassium, systolic blood pressure, blood glucose, AST, dNLR, ABG SpO2, P/F ratio.

As expected, factors identifying respiratory disease severity (WHO disease severity and ABG parameters) were associated with a worse prognosis (Santus et al., 2020). Other factors such as serum potassium concentration, low systolic blood pressure, higher blood glucose and AST values, and heart rate probably reflect acute organ/system failure, while age is a well-established risk factor for adverse outcomes in COVID-19 patients (Henkens et al., 2022).

One of the most relevant findings of our analysis is that mobility level before hospitalization was the only pre-morbid and the most important factor associated with in-hospital mortality. To the best of our knowledge, no other studies have investigated this association in SARS-CoV-2 older patients.

Mobility before hospitalization might be considered as a proxy of preadmission functional status and disability. Indeed, impaired activities of daily living have been shown to be associated with COVID-19 negative outcomes (Ramos-Rincon et al., 2021; Wang et al., 2022; Rodriguez-Sanchez et al., 2021; Laosa et al., 2020; Cangiano et al., 2020; Bruno et al., 2022). However, previous studies did not always address a population of very old adults, had a smaller sample size, or did not include a large set of variables. Moreover, although tools to assess functional status, such as the Barthel Index, do not require formal training, examiners need to be familiar with the functional item being assessed and the scoring system used. Instead, pre-morbid mobility is a piece of information easy to collect by any health personnel involved in the patient’s care.

A second finding of our study is that, in line with previous reports, elevated neutrophil and reduced lymphocyte counts, as reflected by the dNLR index, are important predictors of in-hospital mortality in older inpatients with SARS-CoV-2. While less impactful than other factors, the dNLR index still significantly contributed to a model encompassing variables related acute respiratory and organ dysfunction (Olivieri et al., 2022). Although neutrophils play a role in viral clearance, i.e., with the production of Interferon, they may favor the pathogenesis of SARS-CoV-2 and exacerbate its complications such as acute respiratory distress syndrome (ARDS), thrombosis, and multisystem inflammatory disease (McKenna et al., 2022) and strong evidence has been accumulated on the key role of neutrophils in severe COVID-19 pathogenesis (Zeng et al., 2021; Wang et al., 2020; Wan et al., 2020; Vafadar Moradi et al., 2021; Seyit et al., 2021; Masso-Silva et al., 2022; Ma et al., 2021; Liu et al., 2020; Li et al., 2020; Huang et al., 2020). Elevated neutrophils were found in the nasal epithelium, the lower respiratory tract, and the bloodstream in patients with SARS-CoV-2 infection (Reusch et al., 2021). Moreover, several researches showed that, in severe COVID-19 patients, neutrophils are not only abundant but also have an altered phenotype and functionality (McKenna et al., 2022; Reusch et al., 2021). In particular, increased production of neutrophil extracellular traps (NETs) has been found, with possible direct damage to the pulmonary endothelium and facilitation of the thrombosis pathway, as well as a greater presence of the neutrophil subtype responsible for the suppression of the adaptive immune response, usually typical of a chronic condition such as cancer (McKenna et al., 2022; Reusch et al., 2021).

### Study limitations and strengths

Some limitations of this study should be acknowledged. Data were collected during the first and second COVID-19 waves. This implies that patients were unvaccinated, and variants of SARS-CoV-2 involved in the patients’ infection were different from those currently circulating in terms of the degree of infectivity, the ability to evade the immune response, and the severity of the disease caused.

The present analysis considered only in-hospital mortality, with no information on long-term mortality (or other long-term outcomes), although the occurrence of long-term sequelae of SARS-CoV-2 has also been widely documented.

We did not compare findings deriving from the application of machine learning algorithms with predictions based on conventional statistical methods. However, machine learning models are generally characterized by an overall better, or at least non-inferior, predictive capacity, also concerning COVID-19 outcomes (Casas-Rojo et al., 2023), and most of the predictors included in the model were previously extensively characterized for their prognostic role.

On the other hand, it should be recognized that although many studies have focused on specific aspects associated with SARS-CoV-2 disease-related mortality, only a few have comprehensively assessed a large number of factors in older inpatients, such as our study. Moreover, we considered only variables routinely collected in a hospital setting. This is particularly important since identifying clinical and laboratory parameters among those routinely collected can guide the physician in the early patient risk stratification, facilitating the assessment of the most appropriate care setting and an optimal allocation of health resources.

## Conclusion

Three main conclusions can be drawn from our study. First, in a multivariable analysis encompassing all sociodemographic, acute clinical and laboratory findings, and comorbidities, mobility emerged as the strongest predictor and the only pre-morbid condition that could substantially influence in-hospital mortality in SARS-CoV-2 older adults. Second, the significant feature importance of dNLR that we observed in our model strongly confirms that in SARS-CoV-2 infection, unlike other viral infections, neutrophils play a fundamental role in the pathogenesis and worsening of COVID-19. In this context, dNLR could represent a feasible and inexpensive biomarker of COVID-19 severity in hospitalized older adults. Third, the endpoint of in-hospital mortality can be predicted with good accuracy at the time of admission using functional status indicators and commonly available laboratory results.

These findings support the application of machine learning to develop predictive algorithms based on existing clinical and laboratory variables and highlight the importance of functional status above all other chronic diseases and conditions as a synthetic health measure in older adults, which may be a strong predictor of adverse outcomes.

## Data Availability

The raw data supporting the conclusions of this article will be made available by the corresponding author on reasonable request. Requests to access the datasets should be directed to AC, a.cherubini@inrca.it.

## GeroCovid Observational Working Group

Angela Marie Abbatecola [ASL Frosinone; RSA INI Città Bianca, Veroli (FR)], Domenico Andrieri [RSA Villa Santo Stefano, S. Stefano di Rogliano (CS)], Sara Antenucci [Ambulatorio Psicogeriatrico, Ortona (CH)], Rachele Antognoli (Azienda Ospedaliero Universitaria Pisana; RSA Villa Isabella, Pisa), Rafaele Antonelli RI (Università Campus Bio-Medico, Roma), Maria Paola Antonietti (Ospedale Regionale di Aosta), Viviana Bagalà (Azienda OspedalieroUniversitaria di Ferrara), Giulia Bandini (USL Toscana Centro, Ospedale San Jacopo, Pistoia), Salvatore Bazzano [ULSS 3 Serenissima, Presidio di Dolo (VE)], Giuseppe Bellelli (Ospedale San Gerardo, Monza), Andrea Bellio (Azienda Ospedale Università di Padova), Federico Bellotti (Azienda Ospedaliero-Universitaria di Ferrara), Enrico Benvenuti [USL Toscana Centro, Ospedale Santa Maria Annunziata, Bagno a Ripoli (FI)], Marina Bergamin (Azienda Ospedaliero-Universitaria di Parma), Marco Bertolotti (Azienda Ospedaliero-Universitaria di Modena), Carlo Adriano Biagini (USL Toscana Centro, Ospedale San Jacopo, Pistoia), Angelo Bianchetti (Istituto Clinico Sant’Anna, Brescia), Alessandra Bianchi [Spedali Civili, Montichiari (BS)], Mariangela Bianchi (Policlinico Sant’Orsola-Malpighi, Bologna), Paola Bianchi (Associazione Nazionale Strutture Territoriali e per la Terza Età, Roma), Francesca Biasin (Azienda Ospedale Università di Padova), Silvia Bignamini (Casa di Cura San Francesco, Bergamo), Damiano Blandini (Policlinico Sant’Orsola-Malpighi, Bologna), Stefano Bofelli (Fondazione Poliambulanza, Brescia), Cristiano Bontempi (Azienda Ospedale Università di Padova), Alessandra Bordignon (Azienda Ospedale Università di Padova), Luigi Maria Bracchitta (ATS Milano), Maura Bugada (Casa di Cura San Francesco, Bergamo), Carmine Cafariello [RSA Villa Sacra Famiglia, IHG, Roma; I RSA Geriatria, IHG, Guidonia (RM); III RSA Geriatria, IHG, Guidonia (RM); RSA Estensiva, IHG, Guidonia (RM); RSA Intensiva, IHG, Guidonia (RM)], Veronica Caleri (USL Toscana Centro, Ospedale San Jacopo, Pistoia), Valeria Calsolaro (Azienda Ospedaliero Universitaria Pisana; RSA Villa Isabella, Pisa), Donatella Calvani (USL Toscana Centro, Presidio Misericordia e Dolce, Prato; USL Toscana Centro, Ospedale Santo Stefano, Prato), Francesco Antonio Campagna [Centro di Riabilitazione San Domenico, Lamezia Terme (CZ)], Andrea Capasso (ASL Napoli 2 Nord), Sebastiano Capurso [RSA Bellosguardo, Civitavecchia (RM)], Silvia Carino [RSA San Domenico, Lamezia Terme (CZ); Centro di Riabilitazione San Domenico, Lamezia Terme (CZ); RSA Villa Elisabetta, Cortale (CZ); Casa Protetta Madonna del Rosario, Lamezia Terme (CZ)], Elisiana Carpagnano (Ospedale Giovanni XXIII Policlinico di Bari), Barbara Carrieri (IRCCS INRCA, Ancona), Viviana Castaldo (Presidio Ospedaliero Universitario Santa Maria della Misericordia, Udine), Manuela Castelli [Istituto Geriatrico Camillo Golgi, Abbiategrasso (MI)], Manuela Castellino [Fatebenefratelli, Presidio Ospedaliero Riabilitativo “Beata Vergine Consolata”, San Maurizio Canavese (TO)], Alessandro Cavarape (Presidio Ospedaliero Universitario Santa Maria della Misericordia, Udine), Ilaria Cazzulani (Ospedale San Gerardo, Monza), Carilia Celesti (Policlinico Universitario Campus Bio-Medico, Roma), Chiara Ceolin (Azienda Ospedale Università di Padova), Maria Giorgia Ceresini (Azienda Ospedaliero-Universitaria di Ferrara), Arcangelo Ceretti [Istituto Geriatrico Camillo Golgi, Abbiategrasso (MI)], AC (IRCCS INRCA, Ancona), Anita Chizzoli (Istituto Clinico Sant’Anna, Brescia), Erika Ciarrocchi (IRCCS INRCA, Ancona), Paola Cicciomessere (Azienda Ospedaliero Universitaria di Foggia), Alessandra Coin (Azienda Ospedale Università di Padova), Mauro Colombo [Istituto Geriatrico Camillo Golgi, Abbiategrasso (MI)], Annalisa Corsi (USL Toscana Centro, Ospedale San Jacopo, Pistoia), Antonella Crispino [RSA Villa Santo Stefano, S. Stefano di Rogliano (CS); RSA Villa Silvia, Altilia Grimaldi (CS)], Roberta Cucunato [RSA Villa Santo Stefano, S. Stefano di Rogliano (CS); RSA Villa Silvia, Altilia Grimaldi (CS)], Carlo Custodero (Ospedale Giovanni XXIII Policlinico di Bari), Federica D’Agostino [Casa Sollievo della Sofferenza, S. Giovanni Rotondo (FG)], Maria Maddalena D’Errico [Casa Sollievo della Soferenza, S. Giovanni Rotondo (FG)], Ferdinando D’Amico [RSA San Giovanni di Dio, Patti (ME); RSA Sant’Angelo di Brolo (ME)], Aurelio De Iorio (Azienda OspedalieroUniversitaria di Parma), Alessandro De Marchi [Policlinico Sant’Orsola-Malpighi, Bologna), Annalaura Dell’Armi (III RSA Geriatria, IHG, Guidonia (RM)], Marta Delmonte (Azienda OspedalieroUniversitaria di Ferrara), Giovambattista Desideri [Ospedale di Avezzano (AQ)], Maria Devita (Azienda Ospedale Università di Padova), Evelyn Di Matteo (Policlinico Universitario Campus Bio-Medico, Roma), Emma Espinosa [Azienda Ospedali Riuniti Marche Nord, Fano (PU)], Luigi Esposito [Casa Sollievo della Soferenza, S. Giovanni Rotondo (FG)], Chiara Fazio (Azienda Ospedaliero-Universitaria di Parma), Christian Ferro [RSA Sant’Angelo di Brolo (ME)], Chiara Filippini [Spedali Civili, Montichiari (BS)], Filippo Fini (Azienda Ospedale Università di Padova), Lucia Fiore [Casa Sollievo della Sofferenza, S. Giovanni Rotondo (FG)], Serafna Fiorillo [ASP Vibo Valentia; RSA Madonna delle Grazie, Filadelfa (VV); Casa di Riposo Mons. Francesco Luzzi, Acquaro (VV); Casa di Riposo Villa Betania, Mileto (VV); Casa di Riposo Pietro Rosano, Dasà (VV); Casa di Riposo Serena Diocesi, Mileto (VV); Alloggio per Anziani Villa Amedeo, Francavilla Angitola (VV); Casa Albergo Villa Fabiola, Monterosso Calabro (VV); Casa di Riposo Villa Sara, San Nicola da Crissa (VV); Casa di Riposo Don Mottola, Tropea (VV); Casa di Riposo San Francesco, Soriano Calabro (VV); RSA Anziani, Soriano Calabro (VV); Casa di Riposo Suore Missionarie del Catechismo, Pizzo (VV)], Caterina Fontana (Azienda Ospedaliero-Universitaria di Modena), Lina Forte [Ospedale di Avezzano (AQ)], Riccardo Franci Montorzi (Azienda Ospedaliero Universitaria Careggi, Firenze), Carlo SFu (Azienda Ospedaliero Universitaria Careggi, Firenze), SFu (Azienda Ospedaliero Universitaria Careggi, Firenze), Pietro Gareri (ASP Catanzaro), Pier Paolo Gasbarri (Associazione Nazionale Strutture Territoriali e per la Terza Età, Roma), Antonella Giordano (Azienda Ospedaliero Universitaria Careggi, Firenze), Evelina Giuliani [USL Toscana Centro, Ospedale Santa Maria Annunziata, Bagno a Ripoli (FI)], Roberta Granata (RSA Villa Sacra Famiglia, IHG, Roma), Antonio Greco [Casa Sollievo della Soferenza, S. Giovanni Rotondo (FG)], Nadia Grillo [RSA San Domenico, Lamezia Terme (CZ); Casa di Riposo San Domenico, Lamezia Terme (CZ); RSA Villa Elisabetta, Cortale (CZ)], Antonio Guaita [Istituto Geriatrico Camillo Golgi, Abbiategrasso (MI)], Liana Gucciardino (ASP Agrigento), Andrea Herbst (Azienda Ospedaliero Universitaria Careggi, Firenze), Marilena Iarrera [RSA Sant’Angelo di Brolo (ME)], Giuseppe Ielo (Azienda Ospedaliero-Universitaria di Parma), Valerio Alex Ippolito [Casa Protetta Villa Azzurra, Roseto Capo Spulico (CS)], Antonella La Marca [Casa Sollievo della Soferenza, S. Giovanni Rotondo (FG)], Umberto La Porta (Azienda Ospedaliero-Universitaria di Parma), Ilaria Lazzari (Policlinico Sant’Orsola-Malpighi, Bologna), Diana Lelli (Policlinico Universitario Campus Bio-Medico, Roma), Yari Longobucco (Azienda Ospedaliero-Universitaria di Parma), Francesca Lubian (Ospedale di Bolzano), Giulia Lucarelli (Azienda Ospedaliero Universitaria Careggi, Firenze; USL Toscana Centro, Ospedale San Jacopo, Pistoia), Flaminia Lucchini (Azienda Ospedaliero Universitaria Careggi, Firenze), Daniela Lucente [Spedali Civili, Montichiari (BS)], Lorenzo Maestri (Policlinico Sant’Orsola-Malpighi, Bologna), Marcello Maggio (Azienda Ospedaliero-Universitaria di Parma), Paola Mainquà [Azienda Ospedali Riuniti Marche Nord, Fano (PU)], Mariangela Maiotti [Ospedale San Giovanni Battista, Foligno (PG)], Alba Malara [RSA San Domenico, Lamezia Terme (CZ); Casa di Riposo Villa Marinella, Amantea (CS); Casa Protetta Madonna del Rosario, Lamezia Terme (CZ); Casa Protetta Villa Azzurra, Roseto Capo Spulico (CS); Centro di Riabilitazione San Domenico, Lamezia Terme (CZ); RSA Casa Amica, Fossato Serralta (CZ); RSA La Quiete, Castiglione Cosentino (CS); Casa di Riposo San Domenico, Lamezia Terme (CZ); RSA Villa Elisabetta, Cortale (CZ); RSA Villa Santo Stefano, S. Stefano di Rogliano (CS); RSA Villa Silvia, Altilia Grimaldi (CS)], Carlotta Mancini (Azienda Ospedaliero Universitaria Careggi, Firenze), Irene Mancuso [RSA San Giovanni di Dio, Patti (ME)], Eleonora Marelli [Istituto Geriatrico Camillo Golgi, Abbiategrasso (MI)], Alessandra Marengoni [Spedali Civili, Montichiari (BS)], Eleonora Marescalco (Azienda Ospedale Università di Padova), Benedetta Martin [Ospedale di Avezzano (AQ)], Valentina Massa [Casa Sollievo della Soferenza, S. Giovanni Rotondo (FG)], Giulia Matteucci (Azienda Ospedaliero-Universitaria di Ferrara), Irene Mattioli (Azienda Ospedaliero-Universitaria di Ferrara), Liliana Mazza (Policlinico Sant’Orsola-Malpighi, Bologna), Carmela Mazzoccoli (Ospedale Giovanni XXIII Policlinico di Bari), Fiammetta Monacelli (IRCCS Policlinico San Martino, Genova), Paolo Moneti (RSA Villa Gisella, Firenze), FM (Azienda Ospedaliero Universitaria Pisana; RSA Villa Isabella, Pisa), Federica Morellini (Azienda Ospedaliero-Universitaria di Modena), Maria Teresa Mormile (ASL Napoli 2 Nord), Enrico Mossello (Azienda Ospedaliero Universitaria Careggi, Firenze), Chiara Mussi (Azienda OspedalieroUniversitaria di Modena), Francesca Maria Nigro (USL Toscana Centro, Ospedale Santo Stefano, Prato), Marianna Noale (RSA AltaVita, Istituzioni Riunite di Assistenza, Padova), Chukwuma Okoye (Azienda Ospedaliero Universitaria Pisana), Giuseppe Orio (Policlinico Sant’Orsola-Malpighi, Bologna), Sara Osso [RSA La Quiete, Castiglione Cosentino (CS)], Chiara Padovan (AziendaOspedale Università di Padova), Annalisa Paglia (Azienda Ospedaliero Universitaria di Foggia), Giulia Pelagalli (Azienda Ospedaliero Universitaria Careggi, Firenze), Laura Pelizzoni (Policlinico Sant’Orsola-Malpighi, Bologna), Agostino Perri [RSA La Quiete, Castiglione Cosentino (CS)], Maria Perticone [Casa di Riposo Villa Marinella, Amantea (CS)], Giacomo Piccardo (IRCCS Policlinico San Martino, Genova), Alessandro Picci (Presidio Ospedaliero Universitario Santa Maria della Misericordia, Udine), Margherita Pippi [Ospedale San Giovanni Battista, Foligno (PG)], Giuseppe Provenzano (ASP Agrigento), Matteo Pruzzo (IRCCS Policlinico San Martino, Genova), Francesco Rafaele Addamo [RSA San Giovanni di Dio, Patti (ME)], Cecilia Rafaelli (Azienda Ospedale Università di Padova), Francesca Remelli (Azienda Ospedaliero-Universitaria di Ferrara), Onofrio Resta (Ospedale Giovanni XXIII Policlinico di Bari), Antonella Riccardi (Policlinico Sant’Orsola-Malpighi, Bologna), Daniela Rinaldi (Ospedale di Comunità (Camposampiero), Distretto Alta Padovana, ULSS 6 Euganea, Padova), Renzo Rozzini (Fondazione Poliambulanza, Brescia), Matteo Rubino (IRCCS Policlinico San Martino, Genova), Carlo Sabbà (Ospedale Giovanni XXIII Policlinico di Bari), Leonardo Sacco [Casa Sollievo della Soferenza, S. Giovanni Rotondo (FG)], Mariateresa Santoliquido [Casa Sollievo della Soferenza, S. Giovanni Rotondo (FG)], Mariella Savino [Casa Sollievo della Soferenza, S. Giovanni Rotondo (FG)], Francesco Scarso (Azienda Ospedaliero-Universitaria Sant’Andrea, Roma), Giuseppe Sergi (Azienda Ospedale Università di Padova), Gaetano Serviddio (Azienda Ospedaliero Universitaria di Foggia), Claudia Sgarito (ASP Agrigento), Giovanni Sgrò [RSA Istituto Santa Maria del Soccorso, Serrastretta (CZ); RSA San Vito Hospital, San Vito sullo Jonio (CZ); Casa Protetta Villa Mariolina, Montauro (CZ); Casa Protetta Villa Sant’Elia, Marcellinara (CZ)], Chiara Sidoli (Ospedale San Gerardo, Monza), Federica Sirianni [Casa di Riposo Villa Marinella, Amantea (CS)], Vincenzo Solfrizzi (Ospedale Giovanni XXIII Policlinico di Bari), Benedetta Soli (Azienda Ospedaliero-Universitaria di Modena), Debora Spaccaferro [RSA Estensiva, IHG, Guidonia (RM); RSA Intensiva, IHG, Guidonia (RM)], Fausto Spadea [RSA Casa Amica, Fossato Serralta (CZ)], Laura Spadoni [Ospedale San Giovanni Battista, Foligno (PG)], Laura Tafaro (Azienda Ospedaliero-Universitaria Sant’Andrea, Roma), Luca Tagliafco (IRCCS Policlinico San Martino, Genova), Andrea Tedde (Azienda Ospedaliero-Universitaria di Modena), Camilla Terziotti (Azienda Ospedale Università di Padova), Giuseppe Dario Testa (USL Toscana Centro, Ospedale San Jacopo, Pistoia), Maria Giulia Tinti [Casa Sollievo della Soferenza, S. Giovanni Rotondo (FG)], Francesco Tonarelli (USL Toscana Centro, Presidio Misericordia e Dolce, Prato), Elisabetta Tonon (USL Toscana Centro, Ospedale San Jacopo, Pistoia), CT (Ospedale di Comunità (Camposampiero), Distretto Alta Padovana, ULSS 6 Euganea, Padova; Azienda Ospedale Università di Padova), Rita Ursino [I RSA Geriatria, IHG, Guidonia (RM)], Filomena Vella (Azienda Sanitaria Universitaria Giuliano Isontina, Trieste), Maria Villanova (Azienda Ospedale Università di Padova), Aurora Vitali (Azienda Ospedaliero-Universitaria di Ferrara), SV (Azienda Ospedaliero-Universitaria di Ferrara), Francesca Zoccarato (Azienda Ospedale Università di Padova), Sonia Zotti (Policlinico Universitario Campus Bio-Medico, Roma), Amedeo Zurlo (Azienda OspedalieroUniversitaria di Ferrara).

## Abbreviations

WHO, World Health Organization; EU, European Union; NLR, neutrophil-to-lymphocyte ratio; COPD, chronic obstructive pulmonary disease; JADBIO, Just Add Data Bio; BBC-CV, Bootstrap Bias Corrected Cross Validation; CNS, central nervous system; ABG, arterial blood gas bicarbonate; AST, aspartate aminotransferase; dNLR, derived neutrophil-to-lymphocyte ratio; WBC, white blood cells; n.a, not applicable; ARDS, acute respiratory distress syndrome; NETs, neutrophil extracellular traps.
